# Extracellular Heat Shock Protein 70 Increases the Glucocorticoid Receptor and Dual-Specificity Phosphatase 1 via Toll-like Receptor 4 and Attenuates Inflammation in Airway Epithelial Cells

**DOI:** 10.3390/ijms241411700

**Published:** 2023-07-20

**Authors:** Liang Zhou, Lei Fang, Michael Tamm, Daiana Stolz, Michael Roth

**Affiliations:** 1 Pulmonary Cell Research, Department Biomedicine & Clinic of Pneumology, University & University Hospital of Basel, 4031 Basel, Switzerland; liang.zhou@unibas.ch (L.Z.);; 2 Clinic of Pneumology and Pulmonary Cell Research, University Hospital of Basel, 4031 Basel, Switzerland; daiana.stolz@usb.ch

**Keywords:** heat shock protein 70, glucocorticoid receptor, dual-specificity phosphatase 1, asthma, non-steroidal anti-inflammatory effect

## Abstract

Heat shock protein 70 (HSP70) regulates the ligand binding of the glucocorticoid receptor (GR). In asthma patients, heat treatment increased both the GR expression and secretion of extracellular HSP70 (eHSP70) by bronchial epithelial cells (EC). The objective of this study was to assess the effects of eHSP70 on GR expression and the GR-dependent regulation of immune response in human bronchial ECs. Cells were treated with either eHSP70 or transfected with an expression vector for intracellular HSP70 (iHSP70). Ribonucleic acid (RNA) and protein levels were detected by reverse transcriptase-polymerase chain reaction (RT-PCR), Western blotting, and immunofluorescence. Interleukin (IL-6 and IL-8) secretion was determined by enzyme linked immunosorbent assay (ELISA). The overexpression of iHSP70 decreased, while eHSP70 increased GR expression. In addition, eHSP70 increased the expression of the GR target dual-specificity phosphatase 1 (DUSP-1). In doing so, eHSP70 reduced the tumor growth factor (TGF)-β1-dependent activation of extracellular signal-regulated kinase (Erk)-1/2 and cyclic AMP response element binding protein (CREB) and the secretion of IL-6 and IL-8. Blocking the GR or Toll-like receptor 4 (TLR4) counteracted all eHSP70-induced effects. This study demonstrates a novel anti-inflammatory effect of eHSP70 by the signaling cascade of TLR4-GR-DUSP1, which inhibits TGF-β1-activated pro-inflammatory ERK1/2-CREB signaling and cytokine secretion. The findings suggest that eHSP70 might present a novel non-steroidal therapeutic strategy to control airway inflammation in asthma.

## 1. Introduction

The 70 kDa heat shock protein (HSP70s) belongs to a family of ubiquitous molecular chaperones that control protein folding and hormone receptor function [1]. HSP70 contains a nucleotide-binding domain (NBD), a substrate-binding domain (SBD), and a C-terminal tail of variable length [1]. The main functions of intracellular HSP70 (iHSP70) include (i) the folding of newly synthesized proteins [2,3]; (ii) the translocation of polypeptides into mitochondria, chloroplasts, and the endoplasmic reticulum [4,5]; (iii) the assembly and disassembly of protein complexes [6,7]; (iv) the regulation of protein activity; and (v) the assembly of complexes with other signaling proteins and transcription factors, which regulate the function of the glucocorticoid receptor (GR), such as HSP90 folding and chaperonins [7]. HSP70 also plays a role in stress-related processes such as preventing protein aggregation [8,9], protein disaggregation [8,10,11], the refolding of protein [12], and protein degradation [13,14].

HSP70 can be released upon epithelial cell (EC) death or after extreme stress such as bronchial thermoplasty [15,16]. Extracellular HSP70 (eHSP70) has been reported to have dual immune-regulatory roles: (i) it acts as a signaling molecule, which induces the activation of innate immune cells, or (ii) it attenuates inflammatory response [17,18,19]. However, the role of iHSP70 and eHSP70 in the regulation of the GR in bronchial EC has not been investigated.

Compared to controls, the level of eHSP70 in plasma samples from patients with cancer, cardiovascular disease, diabetes, or trauma was upregulated [20,21,22,23]. Others showed that eHSP70 bound to the macrophage lipid raft micro-domain and stimulated antigen phagocytosis, processing, and the major histo-compatibility complex (MHC)-II presentation of antigens [24]. In THP-1 cells, eHSP70 induced the secretion of interleukin (IL)-1α and IL-8 and, thus, increased inflammation [25]. In contrast, eHSP70 had anti-inflammatory effects via stimulating immune-regulatory T cells, resulting in the downregulation of interferon (IFN)-γ and tumor necrosis factor (TNF)-α, while IL-10 was upregulated [26]. Furthermore, eHSP70 bound both Siglec-5 and Siglec-14, thereby delivering anti-inflammatory signals via Siglec-5, but it was pro-inflammatory when interacting with Siglec-14 [27]. Moreover, eHSP70 was upregulated in chronic immune diseases such as rheumatoid arthritis, achieving a protective anti-inflammatory feedback effect rather than causing immune response [28].

The function of the GR, like many signaling proteins, depends on the interaction with HSP90 and HSP70, which both regulate the structure, function, and ligand binding property of the protein [7]. Earlier, it was reported that eHSP72 upregulated GR expression during the acute phase of sepsis [29]. As previously shown, heat therapy in asthma upregulated the expression of GR and the secretion of eHSP70 by bronchial EC [16]. The data implied that eHSP70 may play a role in the regulation of the GR in bronchial ECs. However, the role of eHSP70 for GR function and regulation remains unclear.

Dual-specificity phosphatase 1 (DUSP1) is a downstream signaling protein of the GR [30,31]. DUSP1 is a key regulator for the resolution of inflammation via dephosphorylating mitogen-activated protein kinases (MAPKs) such as extracellular-signal-regulated kinase (ERK), p38, and c-Jun N-terminal kinase (JNK) [32,33,34].

The objective of this study was to compare the effect of eHSP70 to that of iHSP70 on the regulation of the GR in human bronchial ECs. We observed that eHSP70 upregulated the GR and DUSP1 expression and, thus, achieved an anti-inflammatory effect by blocking ERK-CREB (cyclic AMP response element binding protein) signaling and thereby reduced the TGF-β1-stimulated secretion of interleukin (IL)-6 and IL-8.

## 2. Results

### 2.1. Effects of HSP70 on GR Expression

**Figure 1:**
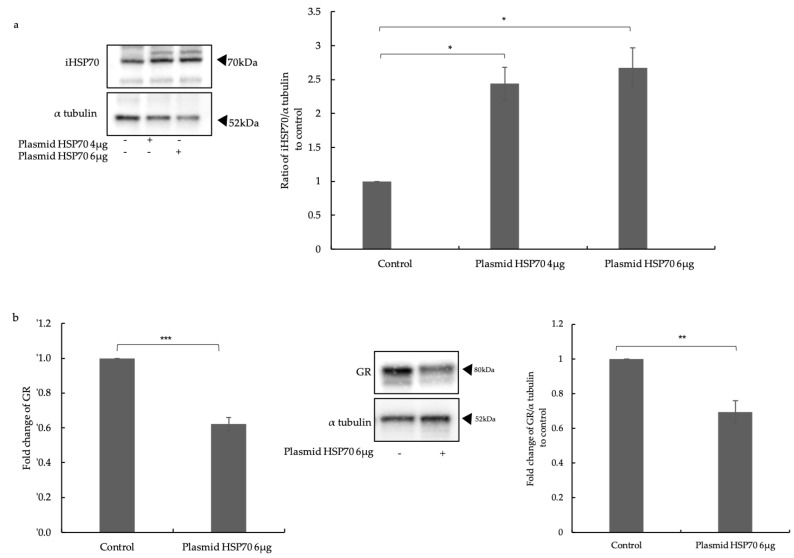
Effect of HSP70 overexpression on the expression of GR. (**a**) Representative Western blots of HSP70 transfection and the image analysis in NuLi-1. Alpha-tubulin served as the house-keeping control protein. Transfection of plasmid HSP70 induced iHSP70 expression in NuLi-1 cells. (**b**) GR mRNA (middle) and protein (right) expression was significantly reduced in cells overexpressing iHSP70. Bars show mean ± S.E.M. of three independent experiments. *p*-values are indicated as follows: * *p* ≤ 0.05; ** *p* ≤ 0.01; *** *p* ≤ 0.001.

The transfection efficiency with the HSP70 expression vector was determined by Western blotting and showed that iHSP70 was significantly upregulated after 24 h (Figure 1a). The quantitative (q)PCR and Western blot analyses showed that the mRNA and protein expression of the GR was significantly downregulated in ECs with high iHSP70 expression (Figure 1b).

**Figure 2:**
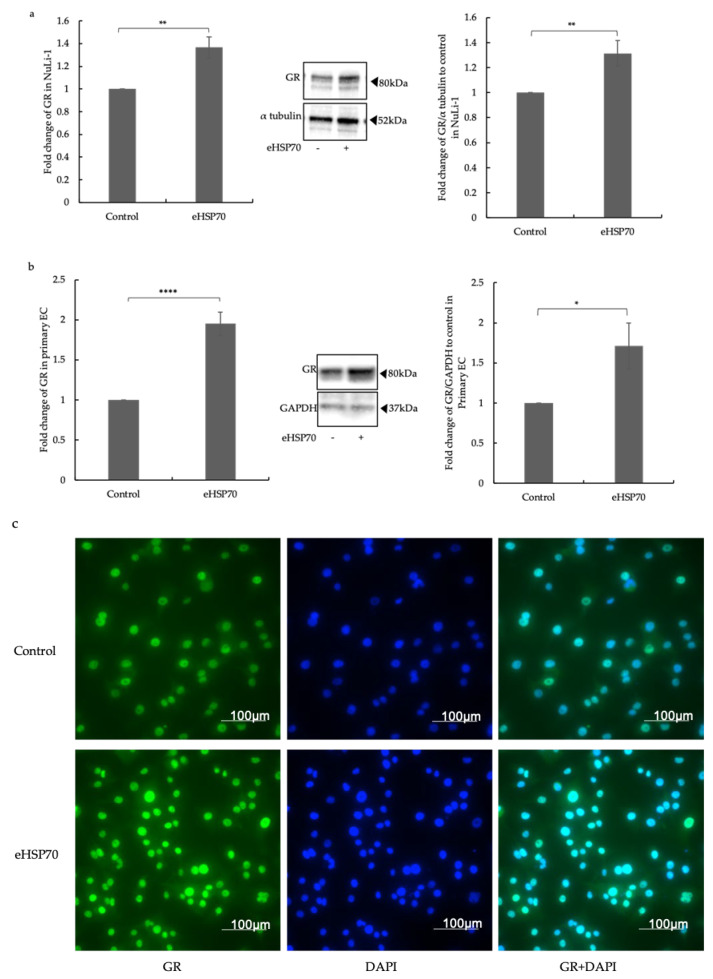
Effects of exposure to eHSP70 on the expression of GR in NuLi-1 and primary ECs. (**a**) Exposure to eHSP70 (5 nM) over 24 h significantly induced the expression of GR mRNA (middle) and protein (right) in NuLi-1. Bars show mean ± S.E.M. of fourteen independent experiments. (**b**) Exposure to eHSP70 (5 nM) significantly induced the expression of GR mRNA (middle) and protein (right) in primary ECs for 24 h. Bars show mean ± S.E.M. of *n* = 5 human primary EC lines. (**c**) Representative photographs show NuLi-1 cells exposed to eHSP70 for 24 h and stained for GR (green) and nuclei (blue); turquoise indicates nuclear GR. *P*-values are indicated as follows: * *p* ≤ 0.05; ** *p* ≤ 0.01; **** *p* ≤ 0.0001.

In contrast, the expression of mRNA and protein of the GR was significantly upregulated after treatment with eHSP70 in both NuLi-1 and primary ECs (Figure 2a,b). Immunofluorescence microscopy showed an increased GR level (green) in NuLi-1 cells after 24 h of treatment with eHSP70 (Figure 2c). Moreover, most of the GR was located in the nucleus, indicating its ligand-independent activation by eHSP70 (Figure 2c). These data indicate that iHSP70 suppressed GR expression in bronchial ECs, while eHSP70 induced GR expression.

### 2.2. Upregulated Expression of DUSP-1

**Figure 3:**
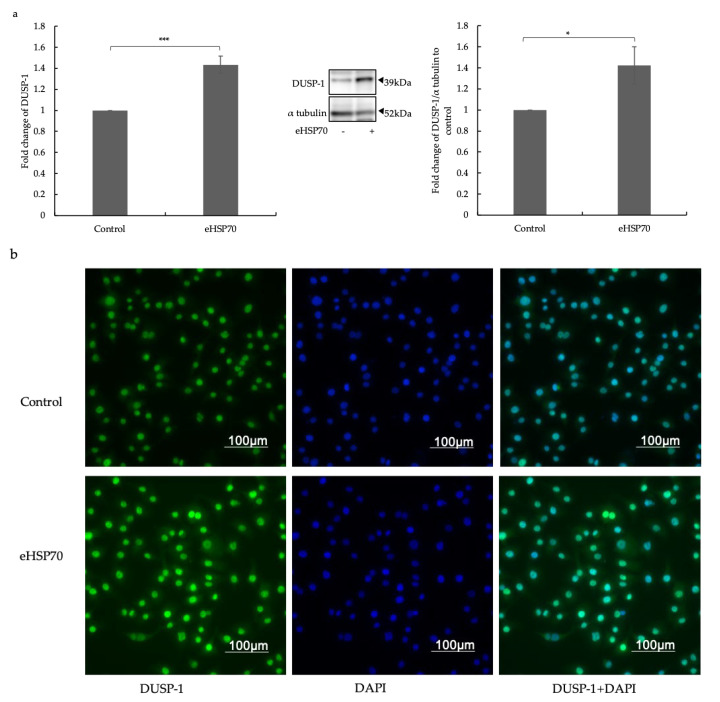
Effects of eHSP70 on the expression of DUSP-1 in NuLi-1 cells. (**a**) Exposure to eHSP70 over 24 h significantly induced DUSP-1 expression in mRNA (middle) and protein (right) in NuLi-1. Bars show mean ± S.E.M. of thirteen independent experiments. (**b**) Representative microphotographs show NuLi-1 exposed to eHSP70 for 24 h and stained for GR (green) and nuclei (blue). *p*-values are indicated as follows: * *p* ≤ 0.05; *** *p* ≤ 0.001.

Next, the expression of the GR target DUSP-1 was compared on the mRNA and protein levels in NuLi-1 cells before and after exposure to eHSP70 (5 mM) over 24 h by Western blot analysis and immunofluorescence. As shown in Figure 3a, DUSP-1 mRNA and protein expression was significantly upregulated after 24 h exposure to eHSP70 in NuLi-1 cells. Immunofluorescence microscopy confirmed this effect of eHSP70 on DUSP-1 expression (green) after 24 h treatment with eHSP70 (Figure 3b). This suggests eHSP70 enhanced DUSP-1 expression in bronchial ECs, presumably as a consequence of the above-described upregulation of the GR by eHSP70.

### 2.3. The Effects of eHSP70 Involve ERK1/2

**Figure 4:**
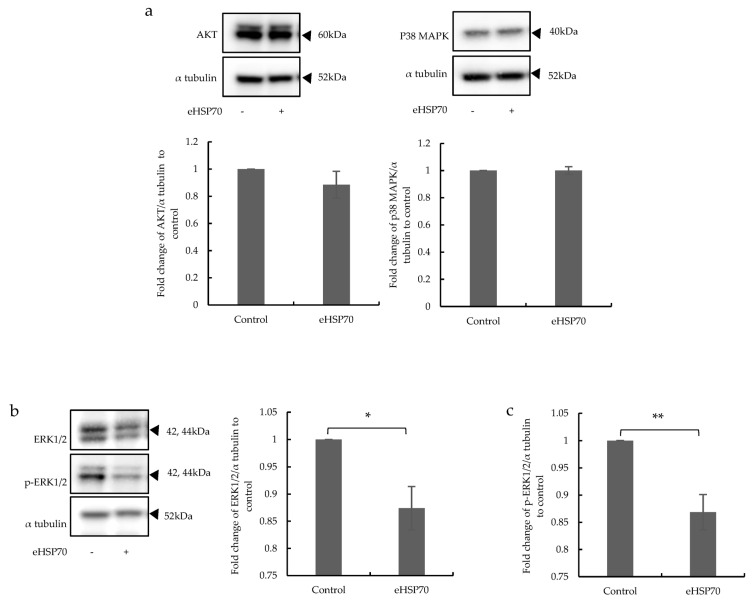
Effects of eHSP70 on the expression of MAPKs in NuLi-1 cells. (**a**) Representative Western blots and bar chart show that exposure to eHSP70 over 24 h did not change the level of AKT (left) or p38 MAPK (right) expression in NuLi-1 cells. Bars show mean ± S.E.M. of three independent experiments. (**b**) Representative Western blots and subsequent image analysis show that eHSP70 significantly reduced ERK1/2 expression and (**c**) phosphorylation of ERK1/2 over 24 h in NuLi-1 cells. Bars show mean ± S.E.M. of five different human primary bronchial EC lines. *p*-values are indicated as follows: * *p* ≤ 0.05; ** *p* ≤ 0.01.

DUSP-1 plays a key anti-inflammatory role via the dephosphorylation of MAPKs. Therefore, the expression and activation of ERK1/2, AKT, and p38 MAPK by Western blot analysis in NuLi-1 cells exposed to eHSP70 (5 nM) for 24 h were assessed. There was no difference in AKT and p38 MAPK expression after exposure to eHSP70 (Figure 4a). However, ERK1/2 MAPK expression was significantly downregulated in eHSP70-treated compared to untreated cells (Figure 4b). Furthermore, the phosphorylation of ERK1/2 was significantly downregulated by eHSP70 (Figure 4c). These results suggest that eHSP70 suppressed the expression and phosphorylation of ERK1/2 MAPK.

### 2.4. Downregulated Expression of CREB

**Figure 5:**
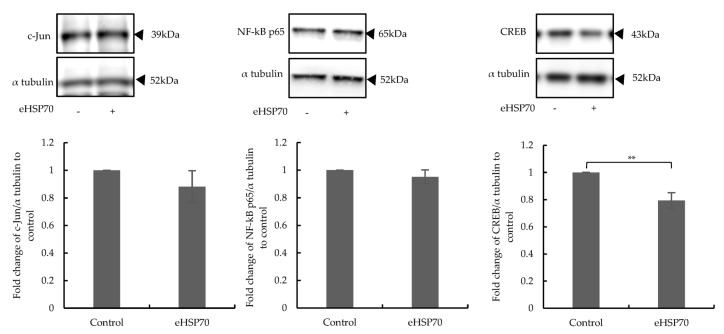
Effects of eHSP70 on the expression of transcription factors in NuLi-1 cells. Exposure to eHSP70 over 24 h did not change c-Jun (left) or NF-kB p65 (middle) expression but reduced that of CREB (right) in NuLi-1 cells. Bars show mean ± S.E.M. of seven independent experiments. *p*-values are indicated as follows: ** *p* ≤ 0.01.

Three transcription factors, CREB, AP-1, and NF-kB, are the main targets of ERK1/2 MAPK [35,36,37]. Therefore, their expressions in NuLi-1 cells after 24 h exposure to eHSP70 (5 nM) was determined by Western blotting. The expression level of CREB was significantly downregulated, while the expression levels of NF-kB and c-Jun were not affected (Figure 5). These data suggest that eHSP70 suppressed CREB as a consequence of the suppression of ERK1/2 by DUSP-1.

### 2.5. Inhibitors of GR and TLRs Prevented the Influence of eHSP70

To provide further information regarding the regulatory effect of eHSP70 on GR expression and activity, NuLi-1 cells were grown for 24 h under five different conditions: (i) culture medium, (ii) eHSP70, (iii) eHSP70 plus GR inhibitor (AL082D06 10 μM), (iv) eHSP70 plus TLR2 inhibitor (C29 100 nM), or (v) eHSP70 plus TLR4 inhibitor (LPS-RS 100 ng/mL).

**Figure 6:**
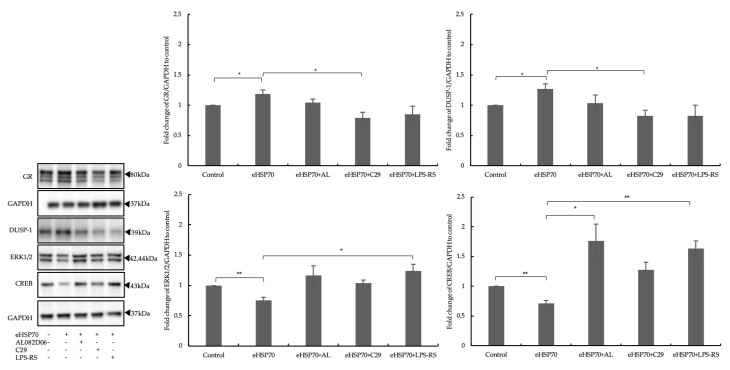
Inhibitors of GR and TLRs prevented eHSP70 effects in NuLi-1 cells. Exposure to eHSP70 over 24 h induced GR (upper left) and DUSP-1 (upper right) expression in NuLi-1 cells. Inhibitors of GR (AL082D06 10 μM), TLR2 (C29 100 nM), or TLR4 (LPS-RS 100 ng/mL) prevented these inductions. In contrast, exposure to eHSP70 over 24 h significantly reduced ERK1/2 (lower left) and CREB (lower right) expression in NuLi-1 cells, which was prevented by inhibitors of GR or TLR. Bars show mean ± S.E.M. of three independent experiments. *p*-values are indicated as follows: * *p* ≤ 0.05; ** *p* ≤ 0.01.

The expressions of GR, DUSP-1, ERK1/2, and CREB were determined by Western blotting. The upregulation of GR and DUSP-1 expression by eHSP70 was mitigated significantly when cells were pre-treated with either the GR inhibitor (AL082D06 10 μM), TLR2 inhibitor (C29 100 nM), or TLR4 (LPS-RS 100 ng/mL) inhibitor (Figure 6). In contrast, the suppression of ERK1/2 MAPK expression and activation by eHSP70 were counteracted in cells treated with either the GR or TLR inhibitors (Figure 6). Thus, these results confirmed that eHSP70 regulates GR and DUSP1 via TLR2 and TLR4.

### 2.6. Cytokines Release

**Figure 7:**
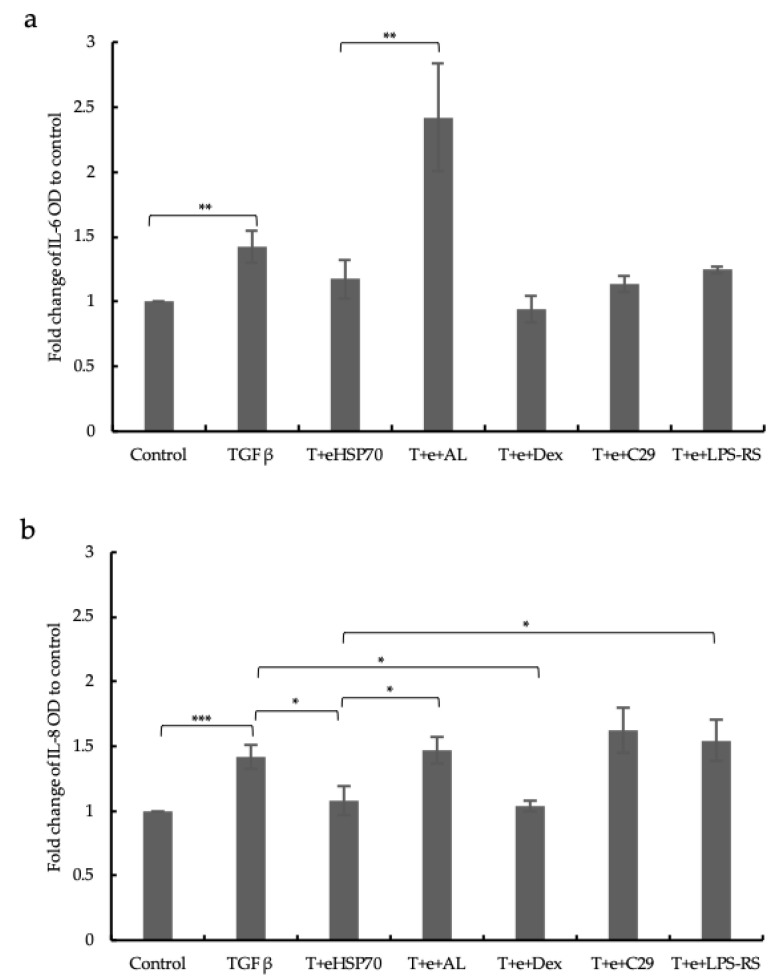
eHSP70 reduces TGF-β1-induced cytokine secretion. TGF-β1 (10 ng/mL) stimulation significantly induced the secretion of IL-6 (upper) and IL-8 (lower) by NuLi-1 cells. Post-stimulative addition of eHSP70 mitigated this effect. GR inhibitor also prevented the eHSP70 effect on both cytokines. The combination of eHSP70 and dexamethasone (10^−8^ M) was used as a control for GR activation. Inhibition of TLR4 (LPS-RS) significantly prevented the negative effect of eHSP70 on IL-8 secretion. T: TGF-β1; AL: AL082D06; e: eHSP70; Dex: dexamethasone. Bars show mean ± S.E.M. of three independent experiments. *p*-values are indicated as follows: * *p* ≤ 0.05; ** *p* ≤ 0.01; *** *p* ≤ 0.001.

Cytokine secretion was determined by ELISA, showing that IL-6 and IL-8 levels were upregulated by TGF-β1 (10 ng/m) in the cell supernatant, which was mitigated by eHSP70 (Figure 7). Furthermore, the levels of IL-6 and IL-8 were significantly higher in cells treated with the GR inhibitor, which might be due to the content of hydrocortisone in the EC culture medium (Figure 7). Furthermore, the treatment with the TLR4 inhibitor prevented the downregulation of IL-8 by eHSP70 significantly (Figure 7b). These results together indicate that eHSP70 downregulates IL-6 and IL-8 via TLR4 and GR.

## 3. Discussion

**Figure 8:**
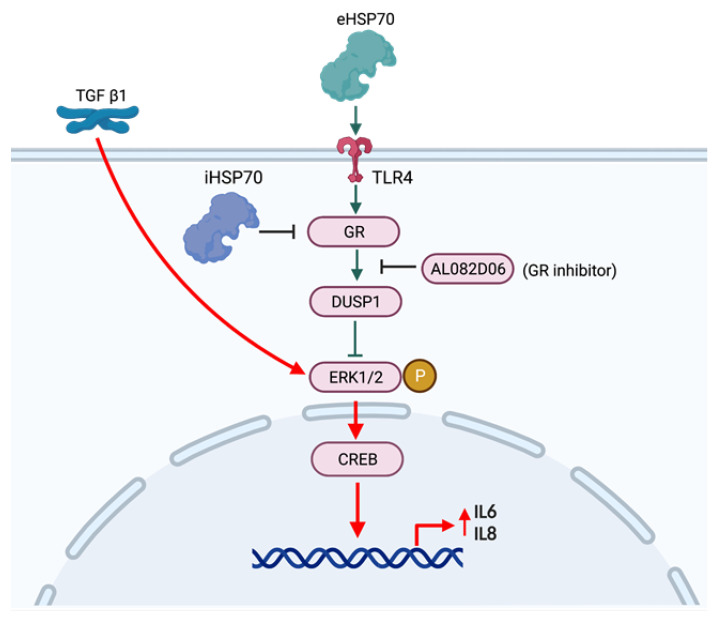
Summary of the anti-inflammatory signaling by eHSP70 via the activation of TLR4, the GR, and DUSP1. Green arrows and lines indicate the anti-inflammatory signaling by eHSP70. Red arrows indicate inflammatory signaling by TGF-β1.

As summarized in the graphic below (Figure 8), the presented data suggest that eHSP70 and iHSP70 have opposing effects on the expression of the GR in human ECs. Furthermore, the data demonstrate a novel anti-inflammatory action of eHSP70, which was mediated by TLR4 and the subsequent increase in GR and DUSP1 expression and activation in ECs. Consequently, the activation of ERK1/2 and CREB was reduced by eHSP70, which also reduced the TGF-β1-stimulated secretion of IL-6 and IL-8.

These results are in line with earlier reports that MAPKs regulated the secretion of IL-6 and IL-8 *by* bronchial ECs [38,39]. TGF-β isoforms have been implicated in the development of chronic airway diseases and regulate pro-inflammatory cytokine release; thus, we used TGF-β1 as model of inflammation [40,41].

HSP70 is mainly regarded as an intracellular protein, which is essential as a chaperone for other proteins and has a specific role in maintaining the ligand binding structure of the GR [1,42]. However, there are increasing reports that HSP70 can be secreted under stress conditions and thereby modulate immune response, inflammation, and tissue remodeling [16,17,19,25,26,27,43].

In vitro, eHSP70 improved the function of bronchial ECs and reduced the proliferation of airway smooth muscle cells [16]. An immunohistological assessment of HSP70 and GR expression in asthma patients treated with bronchial thermoplasty (heat therapy for severe asthma) confirmed cell-type-specific expression in 450 endobronchial biopsies before and after treatment. The expression of both proteins inversely correlated with that of the proliferation marker Ki67, suggesting that high HSP70 and GR expression inhibits the proliferation of airway smooth muscle cells [44]. In line with these findings, clinical studies showed that the use of medication, as well as the expression of inflammation markers, was lastingly reduced after bronchial thermoplasty [45,46,47]. However, the mechanism underlying this lasting effect from bronchial thermoplasty is not fully understood.

Based on earlier studies, eHSP70 was used to treat isolated human airway ECs, which resulted in an upregulation of the GR and the subsequent inhibitor of mitogen-activated protein kinases (MAPKs), DUSP1, also known as MKP1. It has been reported that DUSP1 mediated the anti-inflammatory effect of dexamethasone by blocking the activation of MAPKs [30,31]. The non-lethal heat treatment of immortalized airway epithelial cell lines (BEAS-2B) showed that HSP70 upregulation was followed by the expression of DUSP1 and the downregulation of ERK1/2 and JNK MAPK phosphorylation [48]. In other cell types, the activation of DUSP1 decreased the expression of pro-inflammatory cytokines [32]. In a mouse model, the activation of GR and DUSP1 expression suppressed the secretion of TNF-α, IL-1β, and IL-6 [49]. These observations support the idea that eHSP70 exerts its anti-inflammatory effect via upregulation of the GR and DUSP1.

Downstream of MAPKs, the transcription factor CREB mediated the pro-inflammatory effect of many stimuli, including TGF-β1 [50]. In this study, eHSP70 also downregulated the expression and activation of CREB. The inhibitory effects of eHSP70 on all pro-inflammatory intracellular signaling proteins described above were sensitive to the inhibition of TLR4 and the GR, while TLR2 seems to play a less prominent role. The same applies to the post-stimulatory inhibitory effect of eHSP70 on the TGF-β1-induced secretion of IL-6 and IL-8 reported in this study. The role of TLR4 as a receptor for eHSP70 has been reported in different cell types. In immortalized human embryonic kidney cells, it was reported that TLR4 mediated the pro-inflammatory effect of exogenous HSP70 by activating NF-κB [51]. TLR4 also regulated the growth of endometriosis by HSP70 [52]. In tumor-associated muscle wasting, eHSP70 and TLR4 were identified as major mediators of cell regeneration [53]. The expression level of eHSP70 and its interaction with TLR4 also controlled the regeneration of skeletal muscles in an animal model of muscular dystrophy [54]. In endothelial cells, the interaction between HSP70 and TLR4 protected against oxidative lung injury [55]. Exosomes isolated from plasma contained eHSP70, which protected the myocardium from ischemia–reperfusion injury [56].

The shortfalls of this study are as follows: (i) the lack of a valid animal model, (ii) the investigation of different HSP70 monomers and dimers that regulate its function [57], and (iii) the assessment of heterodimers formed with other HSPs that can regulate its function [58].

In this study, we provide evidence that eHSP70 might reduce the inflammation of ECs by activating GR and DUSP1 expression, thereby reducing the activation of pro-inflammatory MAPK signaling.

## 4. Materials and Methods

### 4.1. Reagents and Antibodies

The antibodies used in this study are listed in Table 1 and Table 2. Recombinant human TGF-β1 (#240-B) and recombinant human HSP70 (eHSP70; #AP-100) were purchased from R&D Systems (Abingdon, UK). The GR inhibitor (AL082D06) was purchased from Selleckchem (#S6608, Houston, TX, USA). The TLR2 inhibitor (C29) was purchased from MedChem Express (#HY-100461, Monmouth Junction, NJ, USA). The TLR4 inhibitor (LPS-RS) was purchased from InvivoGen (#tlrl-rslps, San Diego, CA, USA).

### 4.2. Cell Isolation and Treatment

Primary ECs were obtained from disease-free bronchus sections of resected lung cancer patients (*n* = 5) from the Department of Pathology, University Hospital Basel. The procedure was approved by the local ethics committee (BASEC: PB_2019-00035). Immortalized ECs (NuLi-1) was purchased from ATCC. Primary ECs and NuLi-1 were grown in an epithelial cell selection medium (Cnt-PR-A; CellnTec Advanced Cell Systems, Bern, Switzerland).

Cells were exposed to eHSP70 (5 nM) for 24 h. To provide further information regarding the regulatory effect of eHSP70 on GR expression and activity, NuLi-1 cells were grown in a culture medium under five different conditions for 24 h: (i) culture medium, (ii) eHSP70, (iii) eHSP70 plus GR inhibitor (AL082D06 10 uM), (iv) eHSP70 plus TLR2 inhibitor (C29 100 nM), and (v) eHSP70 plus TLR4 inhibitor (LPS-RS 100 ng/mL).

To prove the effect of eHSP70 on ERK1/2 signaling, NuLi-1 cells were exposed to TGF-β1 (10 ng/mL) 30 min before treatment with either (i) eHSP70, (ii) eHSP70 plus GR inhibitor (AL082D06), (iii) eHSP70 plus TLR2 inhibitor (C29), or (iv) eHSP70 plus TLR4 inhibitor (LPS-RS).

### 4.3. Plasmid Transfection

NuLi-1 cells were transiently transfected with an expression vector for HSP70 (4 μg and 6 μg), which was purchased from OriGene (#RC200270, OriGene Technologies, Inc., Rockville, MD, USA), using Lipofectamine™ 2000 Transfection Reagent (#11668027, Invitrogen™, Waltham, MA, USA) for 6 h. After transfection, cells were cultured in an epithelial cell selective medium, and protein was collected after 48 h.

### 4.4. RNA Extraction and Real-Time Reverse Transcription Polymerase Chain Reaction

Total RNA was isolated using Quick-RNA^TM^ MicroPrep (#R1055, ZYMO RESEARCH, Irvine, CA, USA). Reverse transcription was performed with a High-Capacity cDNA Reverse Transcription Kit (#4368814, Applied Biosystems, Waltham, MA, USA). Quantitative real-time PCR (qPCR) was performed with FastStart™ Universal SYBR^®^(#4913850001, Thermo Scientific, Waltham, MA, USA) to determine the relative gene expression profiles. The primers used for qPCR are listed in Table 3.

Relative mRNA expression was calculated by obtaining the difference between the ΔCt of the target gene from the control group and that of the group under treatment (ΔΔCt). The relative value was expressed as RQ (2^−ΔΔCt^).

### 4.5. Immunofluorescence Staining and Western Blot

Cells were grown in 8-well chamber slides, treated in different conditions, washed with PBS, and then fixed in 4% formaldehyde. Primary antibodies (Table 2) were applied overnight at 4 °C, followed by Alexa Fluor™ 488 (#A-11008, Thermo Scientific). Nuclei were stained by 4′-6-diamidino-2-phenylindole dihydrochloride (DAPI). Images were acquired with ECLIPSE Ti2 (Nikon, Tokyo, Japan) and documented with imaging software NIS-Elements (Nikon).

Cells were lysed in RIPA buffer (#SLCD5849, Sigma, St. Louis, MO, USA), and the protein concentration of each sample was determined by a BCA protein assay kit (#XI357440, Thermo Scientific). The protein concentration was adjusted to standardized levels. For gel electrophoresis, 20 µg of total protein was denatured (10 min at 95 °C), and denatured proteins were size-fractionated (110 V, open Amp, 50 min, at 4 °C) in a 4–12% SDS–PAGE (#M41212, GeneScript, Piscataway, NJ, USA). Proteins were then transferred onto a nitrocellulose membrane (#88018, Thermo Scientific) by heat-accelerated capillary transfer and overnight incubation at 50 °C.

### 4.6. Inhibitor Treatment

The eHSP70-activated intracellular signaling cascade was characterized by pre-incubating the cells with either GR inhibitor (AL082D06, 10 uM), TLR2 inhibitor (C29, 100 nM), or TLR4 inhibitor (LPS-RS, 100 ng/mL) for at least 30 min prior to stimulation with eHSP70.

### 4.7. Enzyme-Linked Immunosorbent Assay

Concentrations of IL-6 and IL-8 in supernatants were detected using a DuoSet ELISA kit (IL-6: DY206, IL-8: DY208,R&D Systems, Abingdon, UK), according to the manufacturer’s instructions.

### 4.8. Statistical Analysis

GraphPad Prism 9.0 software was used for data analysis. Data are represented as mean ± SEM. Statistical analysis was performed by Student’s *t*-test or one-way ANOVA (and nonparametric or mixed) test. The data were presented as mean ± SEM of the results from at least three independent experiments. A *p*-value of <0.05 was considered statistically significant.

## Figures and Tables

**Table 1:** Primary antibodies list used for Western blot analysis.

Primary Antibody	Dilution	Catalog Number	Company
p38 MAPK	1:500	9212S	Cell Signaling (Danvers, MA, USA)
c-Jun	1:1000	ab40766	Abcam (Cambridge, UK)
NF-kB p65	1:400	D14E12	Cell Signaling
HSP70	1:2000	#4873	Cell Signaling
α Tubulin	1:5000	MAB9344	R&D systems
DUSP-1	1:1000	48625	Cell Signaling
ERK 1/2	1:1000	9102l	Cell Signaling
p-ERK1/2	1:1000	9101s	Cell Signaling
AKT	1:1000	4691s	Cell Signaling
CREB	1:1000	#4820	Cell Signaling
GR	1:1000	ab183127	Abcam
GAPDH	1:1000	ab181602	Abcam

**Table 2:** Primary antibodies used for immunofluorescence.

Primary Antibody	Dilution	Catalog Number	Company
DUSP-1	1:100	48625	Cell Signaling
GR	1:500	ab183127	Abcam

**Table 3:** Primer sequences used for real-time reverse transcription.

Gene	Forward Primer (5′-3′)	Reverse Primer (3′–5′)
α tubulin	AGGAGTCCAGATCGGCAATG	GTCCCCACCACCAATGGTTT
hDUSP1	CTGCCTTGATCAACGTCTCA	CTGTGCCTTGTGGTTGTCCT
GR	ATAGCTCTGTTCCAGACTCAACT	TCCTGAAACCTGGTATTGCCT

## Data Availability

All original data can be requested from the first author.
